# Identification of potential plasma protein biomarkers for bipolar II disorder: a preliminary/exploratory study

**DOI:** 10.1038/s41598-021-88450-x

**Published:** 2021-05-04

**Authors:** Sheng-Yu Lee, Tzu-Yun Wang, Ru-Band Lu, Liang-Jen Wang, Sung-Chou Li, Chi-Ying Tu, Cheng-Ho Chang, Yung-Chih Chiang, Kuo-Wang Tsai

**Affiliations:** 1 Department of Psychiatry, Kaohsiung Veterans General Hospital, Kaohsiung, Taiwan; 2 Department of Psychiatry, Faculty of Medicine, Kaohsiung Medical University, Kaohsiung, Taiwan; 3 Department of Psychiatry, College of Medicine, National Cheng Kung University Hospital, National Cheng Kung University, Tainan, Taiwan; 4 Yanjiao Furen Hospital, Hebei, China; 5 Department of Child and Adolescent Psychiatry, Kaohsiung Chang Gung Memorial Hospital and Chang Gung University College of Medicine, Kaohsiung, Taiwan; 6 Genomics and Proteomics Core Laboratory, Department of Medical Research, Kaohsiung Chang Gung Memorial Hospital and Chang Gung University College of Medicine, Kaohsiung, Taiwan; 7 Department of Medical Education and Research, Kaohsiung Veterans General Hospital, Kaohsiung, Taiwan; 8 Department of Research, Taipei Tzu Chi Hospital, The Buddhist Tzu Chi Medical Foundation, New Taipei, 23142 Taiwan

**Keywords:** Neuroscience, Biomarkers, Diseases, Medical research, Molecular medicine, Pathogenesis

## Abstract

The diagnostic peripheral biomarkers are still lacking for the bipolar II disorder (BD-II). We used isobaric tags for relative and absolute quantification technology to identify five upregulated candidate proteins [matrix metallopeptidase 9 (MMP9), phenylalanyl-tRNA synthetase subunit beta (FARSB), peroxiredoxin 2 (PRDX2), carbonic anhydrase 1 (CA-1), and proprotein convertase subtilisin/kexin type 9 (PCSK9)] for the diagnosis of BD-II. We analysed the differences in the plasma levels of these candidate proteins between BD-II patients and controls (BD-II, n = 185; Controls, n = 186) using ELISA. To establish a diagnostic model for the prediction of BD-II, the participants were divided randomly into a training group (BD-II, n = 149; Controls, n = 150) and a testing group (BD-II, n = 36; Controls, n = 36). Significant increases were found in all five protein levels between BD-II and controls in the training group. Logistic regression was analysed to form the composite probability score of the five proteins in the training group. Receiver-operating characteristic curve analysis revealed the diagnostic validity of the probability score [area under curve (AUC) = 0.89, *P* < 0.001]. The composite probability score of the testing group also showed good diagnostic validity (AUC = 0.86, *P* < 0.001). We propose that plasma levels of PRDX2, CA-1, FARSB, MMP9, and PCSK9 may be associated with BD-II as potential biomarkers.

## Introduction

BD-II is a common subtype of bipolar disorder (BD), which has received increasing attention in the past decades^1^. However, diagnosing BD-II is clinically challenging, with an initial misdiagnosis rate of approximately 40% and a 10-year lag of treatment^2^. It is because (1) the first episodes are usually depressive; (2) depressive episodes dominate the course of illness; (3) patients usually request medical attention only during depressive episodes whereas hypomanic episodes are considered as a normal positive mood state^3–5^. Misdiagnosis of BD-II may result in more depressive or hypomanic episodes, less asymptomatic duration, and an increased suicide rate. Besides all these pressures, the socioeconomic environment also burdens the patient^3,6^. Therefore, an accurate and timely diagnosis of BD-II is necessary to enable a further treatment plan and prognosis^7^.

Currently, the diagnosis of BD-II is solely dependent on the clinical presentation and history of the patient. Identification of reliable and clinically applicable biomarkers for BD-II would contribute to prompt diagnosis that can lead to timely treatment. Some peripheral protein markers have been proposed as candidates that differentiate BD from controls^8,9^. However, most of the previous studies only assessed undifferentiated BD and not specific BD-II^10^. The search for candidate peripheral protein biomarkers for BD-II is still inexplicit and warrants further research. Furthermore, the development of a diagnostic tool that provides better accuracy may accomplish “precision psychiatry”^11^.

Proteomics, which quantitatively analyses protein expression in biosamples, has been regarded as a helpful way to identify novel biomarkers, as proteins may assist in the recognition of the underlying pathophysiology^12^. The technology, isobaric tags for relative and absolute quantification (iTRAQ), has been reported to improve the accuracy of protein identification and quantification by reducing the variation in multiple mass spectrometry runs^13^. iTRAQ may therefore be a suitable tool to assist in the identification of protein biomarkers for BD-II. Although previous studies have implemented iTRAQ to compare undifferentiated BD with healthy individuals, so far no study has been done specifically for BD-II^14^.

## Aim of the study

In the present study, we aimed to identify candidate plasma proteins associated with BD-II using iTRAQ in an initially small group of participants. We further explored the association of the identified candidate protein with BD-II in a larger sample. In addition, we plan to evaluate whether these proteins could be plasma biomarkers or form a diagnostic model to assist in BD-II diagnosis. To examine the validity of the diagnostic model using candidate proteins, the participants (BD-II and controls) were divided into training and testing groups.

## Results

We recruited 185 patients with BD-II and 186 controls. Categories of mood states were based on clinical evaluation according to the HAMD and YMRS rating scales without applying duration criteria: Euthymic (HAMD-17 and YMRS < 8), depressive (HAMD-17 > 8 and YMRS < 8), hypomanic (YMRS > 7 and HAMD-17 < 8) and mixed state (HAMD-17 > 7 and YMRS > 7)^15^. Of all patients, 23 were in depressive states, 16 were in hypomanic state; 138 were in mixed state and 8 were in euthymic state. In order to build a diagnostic model using the identified proteins, all participants were randomly selected into the training and testing groups using SPSS. We selected 36 participants from the BD-II and control groups (about 20% of all recruited participants), as the testing group, 80% of the participants were left in the training group. Table 1 showed the clinical characteristics of all participants, listed as training and testing groups. Table 1 shows the clinical characteristics of all participants, listed as training and testing groups. All the recruited patients were first diagnosed with BD-II with no prior treatment for bipolar disorder. All patients were recruited in the morning, and blood samples were collected between 9 am and noon. We did not restrict certain mood states as inclusion criteria. At inclusion, the mean HAMD score was 12.7 ± 4.2; the YMRS score was 11.9 ± 3.6. The mean age of onset of BD-II was 15.3 ± 5.7 years.

**Table 1:** Clinical and demographic data of patients with bipolar II disorder (BD-II) and healthy controls.

	Training cohort				Testing cohort			
	BD-II	Healthy controls	Χ^2^ or t	*P* value	BD-II	Healthy controls	Χ^2^ or t	*P* value
n	149	150			36	36		
Age (mean, SD)	36.1 ± 12.2	32.3 ± 9.0	3.1	0.002*	34.7 ± 12.2	31.8 ± 8.7	1.1	0.27
Gender (M/F)	55/94	82/68	9.5	0.003**	16/30	17/14	3.0	0.10
Education (year)	14.0 ± 2.5	15.4 ± 1.9	− 5.0	< 0.001**	14.1 ± 2.6	15.8 ± 1.5	− 3.2	0.002**
Onset age (year)	15.3 ± 5.7	N/A			15.5 ± 3.9	N/A		
HAMD	12.7 ± 4.2	N/A			13.1 ± 4.6	N/A		
YMRS	11.9 ± 3.6	N/A			10.9 ± 3.3	N/A		
PRDX2 (ng/mL)^a^	13.4 ± 14.2	9.0 ± 9.4	2.6	0.01*	8.4 ± 9.6	7.6 ± 5.6	0.4	0.66
CA-1 (ng/mL)^a^	2434.3 ± 1535.3	1910.6 ± 1512.1	4.4	< 0.001**	2950.4 ± 2970.5	1787.6 ± 1033.1	2.2	0.03*
FARSB (ng/mL)^a^	22.7 ± 12.3	16.6 ± 10.2	5.3	< 0.001**	20.6 ± 10.7	15.0 ± 7.5	2.5	0.01*
MMP9 (ng/mL)^a^	103.6 ± 61.2	67.9 ± 45.8	6.9	< 0.001**	105.3 ± 62.8	58.6 ± 36.3	3.9	< 0.001**
PCSK9 (ng/mL)^a^	330.4 ± 180.0	201.0 ± 88.9	7.3	< 0.001**	305.4 ± 132.9	193.8 ± 80.5	4.3	< 0.001**

We used t-test for continuous variables and chi-square test for categorical variables.

*HAMD* Hamilton depression rating scale, *YMRS* Young manic rating scale, *N/A* not available, *SD* standard deviation.

*P < 0.05; **P < 0.01.

^a^Calculated using transformed data (Log10 transformation of original data for normal distribution).

The ELISA results of the training and testing groups showed that all plasma proteins increased significantly in patients with BD-II compared to controls, in the training and testing groups (Table 1), except that significant differences were not found in the PRDX2 level between BD-II and controls in the testing group. In addition, significant differences in PRDX2, CA-1 and MMP9 levels between male and female were found in the BD-II group. In the control group, significant differences in CA-1, FARSB and MMP9 levels were found between different genders (Supplement Table 1). Due to such difference, we have included age and gender as covariates in the logistic regression testing for predictor for BD-II.

The correlation between plasma levels of proteins and mood severity is shown in Table 2. The level of FARSB negatively correlated with the HAMD (*P* = 0.005) and YMRS scores (*P* < 0.001).

**Table 2:** Correlation between level of protein levels and mood severity in BD-II (training group).

	HAMD		YMRS	
	r	*P*	R	*P*
PRDX2^a^	0.06	0.52	0.13	0.17
CA-1^a^	0.06	0.48	− 0.002	0.98
FARSB^a^	− 0.24	0.005	− 0.35	< 0.001**
MMP9^a^	0.03	0.77	− 0.04	0.69
PCSK9^a^	0.02	0.83	0.15	0.09

*HAMD* Hamilton depression rating scale, *YMRS* Young manic rating scale.

r: Pearson’s correlation coefficient.

*P < 0.05; **P < 0.01.

^a^Calculated using transformed data (Log10 transformation of original data for normal distribution).

**Figure 1:**
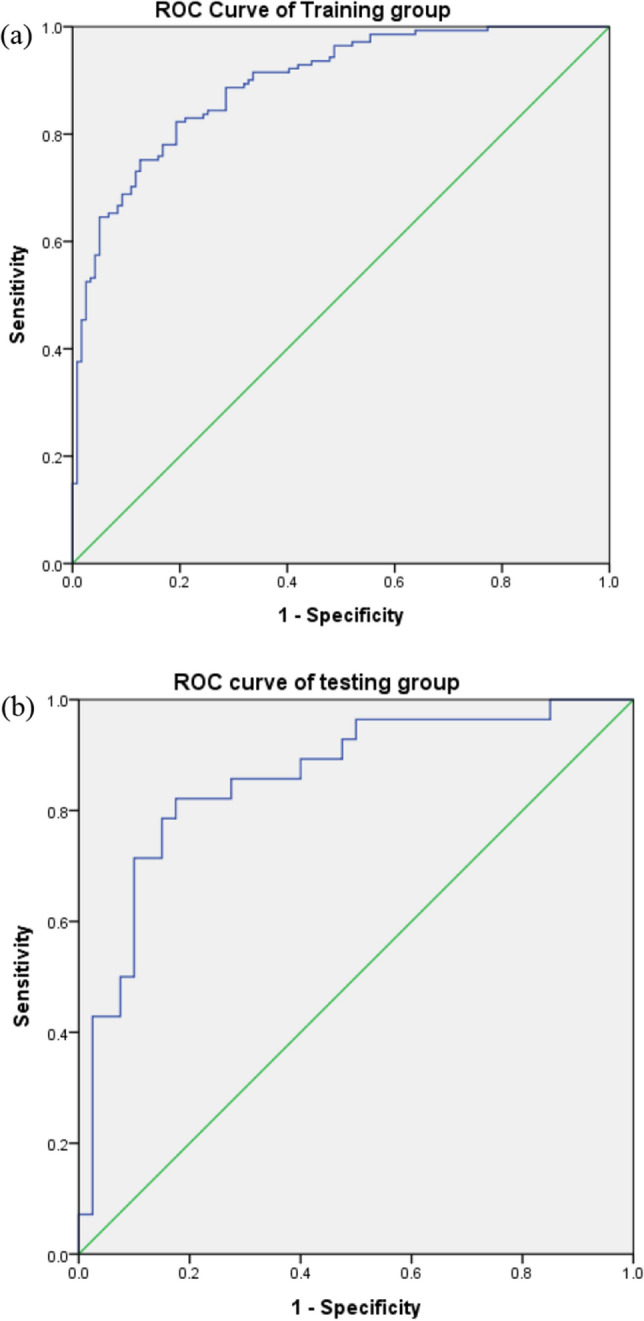
(**a**) The diagnostic power of BD-II of the training group using receiver operating characteristic (ROC) curve analysis. (AUC = 0.89, 95% CI = 0.86–0.93, *P* < 0.001), (**b**) the diagnostic power of BD-II of testing group using receiver operating characteristic (ROC) curve analysis (AUC = 0.86, 95% CI = 0.77–0.91, *P* < 0.001).

Using the training group, we analysed logistic regression to generate a composite probability score (a combination of 5 protein levels) to predict the diagnosis of BD-II (Table 3). We found that the levels of CA-1, FARSB, MMP9, and PCSK9 may predict the diagnosis of BD-II using logistic regression. ROC curve analysis showed that the composite probability score combining MMP9, FARSB, PRDX2, CA-1, and PCSK9 could differentiate BD-II from controls with an AUC of 0.89 (*P* < 0.001, 95% CI = 0.86–0.93) (Fig. 1a).

**Table 3:** Logistic Regression using protein levels to predict diagnosis of BD-II (training group).

	B	95% CI	*P*
PRDX2 (ng/mL)^a^	− 0.005	0.36–2.76	0.992
CA1 (ng/mL)^a^	− 3.6	0.99–1.00	< 0.001**
FARSB (ng/mL)^a^	− 4.0	0.90–0.96	< 0.001**
MMP9 (ng/mL)^a^	− 2.9	0.98–0.99	< 0.001**
PCSK9 (ng/mL)^a^	− 5.2	0.98–0.99	< 0.001**
Gender	1.8	2.2–10.4	< 0.001**
Age	− 0.004	0.96–1.02	0.827

Reference group: normal control.

Constant number: 33.732.

Using logistic regression to form a probability score (combination of 5 protein levels) to predict diagnosis of BD-II.

Probability score for the testing group is calculated in the following way: Logit_probability = 33.732 + (− 0.005) * PRDX2 + (− 3.6) * CA1 + (− 4.0)  * FARSB + (− 2.9)  * MMP9 + (− 5.2) * PCSK9.

Probability score = (2.718281728 ** logit_probability)/(1 + 2.718281728 ** logit_probability).

*P < 0.05; **P < 0.01.

^a^Calculated using transformed data (Log10 transformation of original data for normal distribution).

To replicate the diagnostic validity of the probability score in the aforementioned logistic regression, we further computed the probability score of the five proteins of the testing group using the intercept and B values from logistic regression in Table 3. The ROC analysis of the diagnostic validity of this computed probability score showed that AUC = 0.86 (*P* < 0.001, 95% CI = 0.77–0.91) (Fig. 1b).

Pathway enrichment analysis was conducted for upregulated and downregulated protein candidates, respectively, as shown in Supplementary Figure 2a. The upregulated protein candidates were significantly enriched in critical biological processes, including hydrogen peroxide, cellular oxidant detoxification, and innate immune response. In addition, the downregulated proteins were significantly involved in platelet degranulation, retina homeostasis, actin filament organisation, and muscle filament sliding (Supplementary Figure 2b).

The study had a power of approximately 0.40 to detect a small effect, and 0.99 to detect medium and large effects for independent t-test for the training group (N = 299) by setting a small effect size = 0.2, medium effect size = 0.5, and large effect size = 0.8 and alpha = 0.05^16^. For the regression model, the training group (N = 299) had a power of approximately 0.68 to detect a small effect, and 0.99 to detect medium and large effects by setting a small effect size = 0.02, medium effect size = 0.15, and large effect size = 0.35 (alpha = 0.05)^16^.

## Discussion

In the current study, we identified that plasma levels of PRDX2, CA-1, FARSB, MMP9, and PCSK9 may discriminate patients with BD-II from controls. In addition, the combination of these proteins (MMP9, PCSK9, FARSB, CA-1, and PRDX2) may distinguish BD-II from controls. The identified proteins were never reported to be associated with BD-II in previous studies. Although most of the proteins are related to the inflammatory process, the underlying mechanisms of how these proteins are involved in the pathogenesis of BD-II require further investigation.

PRDX2 is a 2-Cys antioxidant enzyme of the peroxiredoxin family and is abundantly expressed in mammalian cells. It protects cells from oxidative stress because PRDX2 may scavenge levels of H2O2 and ROS^17^. PRDX2 protein is essential for redox balance and prolongs cell lifespan^18^. PRDX2 overexpression has been reported in gastric cancer^18^ and correlated with the progression of colon, cervical, and lung cancers. We are first to report the association between the increase of PRDX2 protein levels and BD-II. Moreover, it may assist with the prediction of BD-II. Further studies are warranted to clarify the underlying mechanism of PRDX2 proteins interaction in BD-II and role of oxidative stress in it.

We also found an increase in the levels of CA-1 and FARSB in BD-II patients compared to controls. CA-1 is an enzyme that catalyses the reversible hydration of carbon dioxide^19^. It is found primarily in the red blood cells, the colonic epithelium, and neutrophils^20^. Association of CA-1 with gastrointestinal inflammation has been derived from previous data that showed decreased CA-1 protein in the inflamed mucosa of ulcerative colitis^21^. Significantly increased expression of CA-1 was found in the aortic lesions, particularly in calcified regions^22^. CA-1 is also proposed as a biomarker for the diagnosis of non-small cell lung cancer as it was found to be highly expressed in serum^23^. Although CA-1 has been never associated with any mental disorder because it was reported to mediate cerebral vascular permeability, its patho-mechanism in BD-II still requires further investigation. FARSB catalyses the synthesis of phenylalanyl-tRNAPhe (Phe-tRNAPhe). Mutation of the FARSB gene leads to a multisystem disease, aminoacyl-tRNA synthetase-related diseases, characterised by the following clinical features: brain calcifications, cerebral aneurysms, interstitial lung disease, and cirrhosis^24^. We found significantly negative correlations between the level of FARSB and clinical symptoms. Therefore, although we found significant association of that the FARSB levels may predict BD-II, the FARSB may be a state but not a trait biomarker. However, since no previous studies have reported how FARSB proteins are expressed in mental disorders, the underlying pathogenesis of FARSB with BD-II requires further study. A trait marker is enduring which may reflect the underlying pathophysiology of the disease, while a state marker reflects clinical manifestation and may be changeable. We found that the FARSB significantly correlated with HAMD and YMRS scores, this rather speaks for a state and not a trait biomarker. Being a state marker, the FARSB may reflect treatment response of BD-II, which is clinically important as well. The underlying mechanism for the correlation between FARSB level and clinical symptoms of BD-II requires further investigation. Because most of the patients recruited in this study were in mixed episode, it will be of interest to explore whether significantly higher levels of FARSB is noted in euthymic group of BD-II compared to controls, which may support its role as a trait marker.

Our finding partially agrees with a previous study that reported increased MMP9 proteins in non-differentiated BD compared to controls; this report suggested that MMP9 is a staging marker^25^. MMP9 may increase the inflow of cytokines into the central nervous system (CNS) by increasing the permeability of the blood–brain barrier (BBB)^26^. The increased inflow may exert neurotoxic and gliotoxic effects, which further contribute to an inflammatory process in the CNS. Since CNS inflammation and neurodegeneration may be involved in the pathogenesis of BD^27^, the current finding of increased plasma MMP9 levels in BD-II may provide a further reference from the inflammation aspect for the association of BD and inflammation.

We found an increase in the plasma levels of PCSK9 in BD-II compared to controls. PCSK9 is a hepatic enzyme that may modulate the metabolism and homeostasis of plasma cholesterol. PCSK9 promotes the degradation receptors of low-density lipoprotein (LDL) and very-low-density lipoprotein (VLDL) by binding to these receptors^28^. So, an increase in the level of PCSK9 may result in the degradation of the receptors of LDL and VLDL, thereby elevating serum LDL-cholesterol levels and increasing the risk for cardiovascular disease^28^. Contrarily, a decrease in PCSK9 level can lower blood LDL-cholesterol concentrations^28^. In mental disorders, higher CSF levels of PCSK9 were found in patients with Alzheimer's Disease^29^ and patients with alcohol use disorder^30^ than in controls. In addition, plasma PCSK9 levels were found to correlate positively with PCSK9 levels in the CSF of patients with alcohol use disorder. Recent studies have suggested a direct relationship between PCSK9 levels and inflammation^31^. It has also been proposed that anti-inflammatory agents may have inhibitory effects against the PCSK9 enzyme^32^. PCSK9 inhibitors have been reported to be effective for neuroprotection with no negative impact on cognition^33^. However, the exact mechanism for the relationship between PCSK9 and BD-II requires further investigation.

We further performed gene ontology analysis of the identified proteins to elucidate the functional relevance with biological processes. The identified pathways of upregulated proteins included regulation of lipoprotein, response to oxidative stress, and innate immune response (Supplementary Figure 2a), which were all previously associated with the pathogenesis of affective disorders^34,35^. However, further mechanistic studies are required to elucidate the exact function of identified proteins and how they interact in the pathogenesis of BD-II.

The major finding of the current study is the identification of individual candidate proteins and the combination of these proteins as diagnostic biomarkers for BD-II. The AUC of the ROC curve was 0.89. This proposed diagnostic panel consisting of five plasma proteins may be an applicable and convenient clinical tool to assist in BD-II diagnosis. This model was further validated by the testing group, which also showed a diagnostic validity of AUC = 0.86. However, whether this model may distinguish BD-II from BD-I or major depressive disorder, which are clinically challenging, still warrants future study that encompasses recruitment of more groups with affective disorders.

Our study has the following shortcomings that should be interpreted with caution. First, it will be ideal to subject the entire sample to iTRAQ analysis for the identification of candidate protein biomarkers. With limited funding, we were unable to afford this expense and only subjected the initial group to iTRAQ analysis. Contrarily, our results would be much more representative if we applied the sampling pool method from each group and then subject the samples to iTRAQ. Our study result is therefore very exploratory and preliminary which requires further confirmation. We only selected upregulated proteins in the current study because we were not sure whether the downregulated candidates were undetectable in BD-II or not. We thought that upregulated protein biomarkers might be easier to detect whereas downregulated proteins may limit detection ability^36^. Hence, we may have neglected candidate proteins whose expression was downregulated in BD-II. In addition, we sampled plasma proteins instead of central nervous system samples (cerebrospinal fluid); therefore, its applicability to other types of samples is unknown. Third, we did not control for some frequently occurring metabolic comorbidities, including diabetes and hypertension, as these diseases may confound the correlation between candidate proteins and BD-II. In addition, we did not control for the time of fasting before blood sampling. As some proteins may change according to age, the results from our relatively young population should be interpreted with caution. Because all participants were randomly selected into the training and testing groups using SPSS, there were significant differences in age and gender between BD-II and controls in the training cohort, but not in the testing cohort. There is a trend of differences in age and gender between BD-II and controls in the testing cohort also; however, the differences were not-significant, probably due to the smaller sample size compared to the training cohort. Future studies with age and gender matched case and control groups in both the training and testing cohorts may be warranted. In the current study, as a control for differences in age and gender, we included gender and age as covariates in the logistic regression model. However, the statistical difference in age and gender between patients and controls may still confound the validity of our results. Only proteins that are not influenced by clinical state or gender differences between patient and controls are really of use as diagnostic markers, therefore, the proteins we identified in the current study still require further validation. Further study focusing on patients in euthymic state may be needed as well to clarify whether FARSB is a trait marker as well. Fourth, as we mentioned earlier, other affective disorders which are frequently confused or misdiagnosed with BD-II, including major depressive disorder and BD-I, were not recruited in the current study as comparative groups. We, therefore, are not sure whether the current model may assist with the common misdiagnosis of BD-II as other mood disorder. Although, we analysed the correlation between protein levels and mood severity, we were unable to evaluate the change of each candidate protein in disease progression or after administration of BD-II treatment due to the cross-sectional design of the current study. It will be ideal for a future longitudinal study to observe the changes in candidate proteins alone in the course of illness to determine whether they are suitable treatment targets.

## Conclusion

We have identified candidate protein biomarkers—PRDX2, CA-1, FARSB, MMP9, and PCSK9—associated with BD-II in the current study. We also found that the combination of these five proteins may predict the diagnosis of BD-II with good validity. We believe that these plasma protein biomarkers may be an addition to precision psychiatry by assisting in the identification and recognition of BD-II. Prompt and accurate diagnosis may facilitate timely pharmacological and psychological intervention, which not only decreases the lengthened and difficult course of the disease but can also alleviate the socioeconomic burden on society.

## Methods

We conducted the study in the Department of Psychiatry, Kaohsiung Veterans General Hospital and National Cheng Kung University Hospital. The institutional review boards of Kaohsiung Veterans General Hospital (VGHKS19-CT5-17) and National Cheng Kung University Hospital (B-BR-108-046) approved the research protocol. The purpose of the study was explained to all participants, and their informed consent was obtained. All methods were performed in accordance with relevant guidelines and regulations.

### Patients and procedures

Diagnosed by research psychiatrists, patients with BD-II aged between 20 and 65 years were recruited from inpatient and outpatient clinics at these two medical centres. The patients further underwent a structural interview, the Chinese version of the Modified Schedule of Affective Disorders and Schizophrenia-Life Time (SADS-L)^37^, to confirm their diagnosis. For diagnosis of hypomanic episodes, we used a 2-day minimum criterion because previous epidemiologic data^38,39^ suggest that a 2-day minimum duration may be more common in community samples than the 4-day duration defined in the Diagnostic and Statistical Manual of Mental Disorders, Fourth Edition Text Revision (DSM-IV-TR; American Psychiatric Association, 2000). The inclusion criterion for recruitment included fitting the diagnosis of BD-II. Exclusion criteria were (a) any other major and minor mental illnesses besides BD-II, such as organic mental disorders and substance use disorder and (b) any significant medical or neurological disorders.

Healthy controls were recruited from the community. These participants also received structural interviews using the SADS-L to screen for psychiatric conditions. Inclusion criteria for the controls were: (a) age between 20 and 65 years; (b) no major or minor mental illnesses (such as schizophrenia, mood disorders, anxiety disorder, substance use disorder, and personality disorder) and no family history of psychiatric disorder among their first-degree relatives; (c) no blood transfusions or severe trauma within the past month.

### Measures of symptomatology

Mood severity was evaluated by research psychiatrists using the Young Mania Rating Scale^40^ and the Hamilton Depression Rating Scale^41,42^.

### Plasma collection

Twenty millilitres of whole blood was collected from the antecubital vein of each participant. Blood samples were collected in a test tube containing ethylenediaminetetraacetic acid (EDTA) (Greiner Bio-One Vacuette; Santa Cruz Biotechnology, Santa Cruz, CA), kept on ice for no more than 30 min. To isolate plasma, whole blood was prepared by centrifuging at 3000 g for 15 min at 4 °C and then stored at − 80 °C for further evaluation.

### iTRAQ library preparation and screen plasma protein

In this study, we randomly selected plasma samples from two BD-II patients and two controls as initial groups for the iTRAQ analysis to identify candidate proteins. Four plasma samples were first subjected to high-abundance protein depletion using the Pierce Top 12 Abundant Protein Depletion Spin Columns (85165, ThermoFisher Science). Then, the protein library of the four plasma samples was prepared using the iTRAQ Reagents Multiplex Kit (4352135, Sciex), and the quality of the library was further confirmed. Finally, the four libraries of plasma samples were analysed with LC/Q-Exactive Orbitrap MS (Thermo) for 24 h. The raw data were analysed with Proteome Discoverer v2.4 (Thermo) by referring to the MASCOT 2.5 database (Matrix Science).

The analysis of labeled peptides was performed using an LTQ Orbitrap Velos ETD mass spectrometer (Thermo Scientific, Bremen) connected to an Agilent 1200 nanoLC system. The online reversed-phase chromatography included a trapping column (75 µm × 2 cm, C18 material 5 µm, 100 Å) with a flow rate of 4 µL/min and an analytical column (75 µm × 10 cm, Magic C18 AQ, 3 µm particle size, pore size 100 Å) with a flow rate of 350 nL/min. The peptide was eluted using a linear gradient of 5 to 45% acetonitrile in 75 min. The electrospray source consisted of a 10 ± 2 µm emitter tip (New Objective, MA, Woburn) maintained at 2.4 kV. The full scan MS acquired with the mass resolution of 60,000 and the MS/MS scan with the mass resolution of 15,000 using the FT mass analyzer were used to collect data independently in a data-dependent manner. For each survey scan in the MS cycle, twenty strongest precursor ions were selected for MS/MS. HCD fragmentation was performed at 42% normalized collision energy. To avoid repeated selection of ions for MS/MS, the dynamic exclusion window was set to 30 s. The AGC settings for the complete FT MS and FT MS/MS were 1 million and 100,000 ions, and the maximum accumulation time were 300 ms. The use of polydimethylcyclosiloxane (m/z, 445.1200025) ion could accurately measure the locked mass. We followed previous research^43^ for these detailed experimental steps.

**Figure 2:**
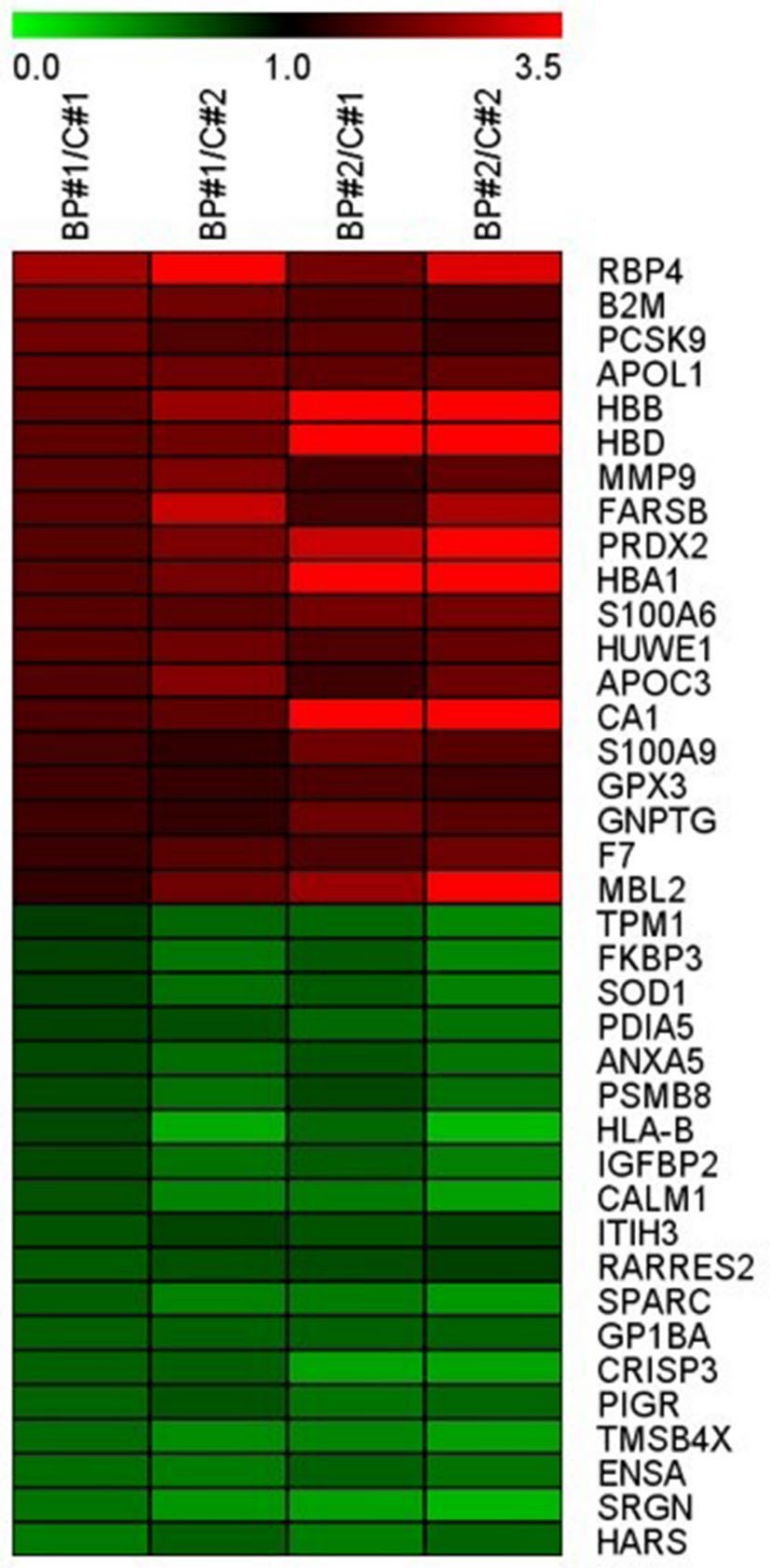
Heatmap of candidate proteins: the top 38 differentially expressed protein candidates (19 with upregulation and 19 with downregulation) presented with heatmap.

From iTRAQ analysis**,** we detected 827 proteins in these samples according to two specific parameters: (1) FDR for protein < 0.01 and peptide identification > 2; (2) the number of protein match unique peptides ≥ 1.5 or ≦ 0.75. The iTRAQ data were further analysed using Partek software (Qiagen, Germany), and the expression of candidate proteins between healthy control and BD-II were analysed by t-test. We identified 49 proteins with significantly increased expression and 88 proteins with significantly decreased expression in BD-II patients compared to those in controls (Supplementary Figure 1). The top 38 differentially expressed protein candidates were presented using a heatmap (19 upregulated and 19 downregulated proteins) (Fig. 2).

From the top 19 upregulated protein candidates, we selected five candidate proteins for further analysis by meeting the following criteria: (1) detection in all samples that showed differential expression twofold relative to the control sample and (2) relation to inflammatory and oxidative stress, as these might be pathogenesis of bipolar disorder^44^. The selected candidate proteins were MMP9^26^, FARSB^45,46^, PRDX2^17^, CA-1^21^ and PCSK9^31^.

### Evaluate concentration of candidate proteins by ELISA

The expression levels of five candidate proteins—MMP9, FARSB, PRDX2, CA-1, and PCSK9—in the plasma of BPII and healthy controls were assessed using ELISA kits. The ELISA kits used in this study were as follows: MMP9 (ARG80129, Arigo, Taiwan), FARSB (EH8444, FineTest, China), PRDX2 (ARG82038, Arigo, Taiwan), CA-1 (ARG82250, Arigo, Taiwan), and PCSK9 (ARG81395, Arigo, Taiwan). An ELISA reader (SpectraMax-M2; Molecular Devices, Sunnyvale, CA), which has a minimum detectable dose of 80 pg/mL, was used.

### Pathway enrichment analysis

These identified proteins were subjected to Gene Ontology analysis using DVVID Bioinformatics Resources 6.8 (PMID: 19131956) to identify significantly enriched pathways.

### Statistical analysis

Because the protein data did not follow a normal distribution but were skewed to the right, we took Log10 for all protein levels to transform the protein data to a normal distribution for further analysis. The *t*-tests and chi-square tests were used to analyse the differences in clinical variables and plasma protein levels between patients and controls. Pearson’s correlation was used to analyse the correlations between the level of plasma proteins and mood severity. Logistic regression was further performed to identify whether each candidate may predict BD-II from controls by setting BD-II as the dependent variable and levels of each plasma protein as independent variables in one model, controlling for age and gender. In addition, we used logistic regression to form a composite probability score of the combination of MMP9, FARSB, PRDX2, CA-1, and PCSK9 protein levels in order to predict the diagnosis of BD-II. By analysing the receiver operating characteristic (ROC) curves and the area under the ROC curve (AUC) of the composite probability scores generated by the above logistic regression, we tried to determine whether the combination of MMP9, FARSB, PRDX2, CA-1, and PCSK9 could distinguish between BD-II and control groups. The cut-off values of optimal diagnostic points of the ROC curve were set at the largest Youden’s index (sensitivity and specificity-1). Using the random sampling method in SPSS, the patients were then divided into training (BD-II, n = 149; control, n = 150) and testing groups (BD-II, n = 36; control, n = 36) in order to conduct the replication study. We further computed the composite probability scores of the testing group using the intercept value and the B values from the logistic regression of the training group. Moreover, we analysed the ROC analysis of the computed composite probability scores of the testing group to examine whether these five proteins may effectively differentiate BD-II from controls. We used the statistical software SPSS v25.0 (Armonk, NY: IBM Corp.) to perform all statistical analyses.

We performed the power analysis using G-Power 3.1.9.2^16,47^, and the effect-size conventions were determined according to Buchner et al.^16^.

## Supplementary information

Supplementary Information 1.

Supplementary Information 2.

## References

[1] Vieta E (2019). Bipolar II disorder: frequent, valid, and reliable. Can. J. Psychiatry.

[2] Manning JS, Haykal RF, Connor PD, Akiskal HS (1997). On the nature of depressive and anxious states in a family practice setting: the high prevalence of bipolar II and related disorders in a cohort followed longitudinally. Compr. Psychiatry.

[3] Ghaemi SN, Boiman E, Goodwin FK (2000). Insight and outcome in bipolar, unipolar, and anxiety disorders. Compr. Psychiatry.

[4] Ghaemi SN, Soldani F, Hsu DJ (2003). Evidence-based pharmacotherapy of bipolar disorder. Int. J. Neuropsychopharmacol. Off. Sci. J. Coll. Int. Neuropsychopharmacol..

[5] Angst J (2007). The bipolar spectrum. Br. J. Psychiatry J. Ment. Sci..

[6] MacQueen GM, Young LT (2001). Bipolar II disorder: symptoms, course, and response to treatment. Psychiatr. Serv..

[7] Suppes T, Hirschfeld RM, Vieta E, Raines S, Paulsson B (2008). Quetiapine for the treatment of bipolar II depression: analysis of data from two randomized, double-blind, placebo-controlled studies. World J. Biol. Psychiatry.

[8] Bartoli F, Carra G, Clerici M (2017). Update on bipolar disorder biomarker candidates: what about uric acid/adenosine hypothesis?. Expert Rev. Mol. Diagn..

[9] Teixeira AL, Salem H, Frey BN, Barbosa IG, Machado-Vieira R (2016). Update on bipolar disorder biomarker candidates. Expert Rev. Mol. Diagn..

[10] Menezes IC, von Werne Baes C, Lacchini R, Juruena MF (2019). Genetic biomarkers for differential diagnosis of major depressive disorder and bipolar disorder: a systematic and critical review. Behav. Brain Res..

[11] Fernandes BS (2017). The new field of 'precision psychiatry'. BMC Med..

[12] Taurines R (2011). Proteomic research in psychiatry. J. Psychopharmacol..

[13] Zieske LR (2006). A perspective on the use of iTRAQ reagent technology for protein complex and profiling studies. J. Exp. Bot..

[14] Ren J (2017). Identification of plasma biomarkers for distinguishing bipolar depression from major depressive disorder by iTRAQ-coupled LC-MS/MS and bioinformatics analysis. Psychoneuroendocrinology.

[15] Zimmerman M, Martinez JH, Young D, Chelminski I, Dalrymple K (2013). Severity classification on the Hamilton Depression Rating Scale. J. Affect. Disord..

[16] G-Power (1996). A Priori, Post Hoc, and Compromise Power Analyses for the Macintosh, v. Version 2.1.1..

[17] De Franceschi L (2011). Oxidative stress modulates heme synthesis and induces peroxiredoxin-2 as a novel cytoprotective response in beta-thalassemic erythropoiesis. Haematologica.

[18] Wang S (2020). PRDX2 protects against oxidative stress induced by *H. pylori* and promotes resistance to cisplatin in gastric cancer. Redox Biol..

[19] Supuran CT (2008). Carbonic anhydrases—an overview. Curr. Pharm. Des..

[20] Torella D (2014). Carbonic anhydrase activation is associated with worsened pathological remodeling in human ischemic diabetic cardiomyopathy. J. Am. Heart Assoc..

[21] Robinson CE (1997). Regulation of neutrophils in ulcerative colitis by colonic factors: a possible mechanism of neutrophil activation and tissue damage. J. Lab. Clin. Med..

[22] Yuan L (2019). Carbonic anhydrase 1-mediated calcification is associated with atherosclerosis, and methazolamide alleviates its pathogenesis. Front. Pharmacol..

[23] Wang DB, Lu XK, Zhang X, Li ZG, Li CX (2016). Carbonic anhydrase 1 is a promising biomarker for early detection of non-small cell lung cancer. Tumour Biol..

[24] Xu Z (2018). Bi-allelic mutations in Phe-tRNA synthetase associated with a multi-system pulmonary disease support non-translational function. Am. J. Hum. Genet..

[25] Reininghaus EZ (2016). Extracellular matrix proteins matrix metallopeptidase 9 (MMP9) and soluble intercellular adhesion molecule 1 (sICAM-1) and correlations with clinical staging in euthymic bipolar disorder. Bipolar Disord..

[26] Turner RJ, Sharp FR (2016). Implications of MMP9 for blood brain barrier disruption and hemorrhagic transformation following ischemic stroke. Front. Cell Neurosci..

[27] Fries GR, Walss-Bass C, Bauer ME, Teixeira AL (2019). Revisiting inflammation in bipolar disorder. Pharmacol. Biochem. Behav..

[28] Urban D, Poss J, Bohm M, Laufs U (2013). Targeting the proprotein convertase subtilisin/kexin type 9 for the treatment of dyslipidemia and atherosclerosis. J. Am. Coll. Cardiol..

[29] Zimetti F (2017). Increased PCSK9 cerebrospinal fluid concentrations in Alzheimer's disease. J. Alzheimers Dis..

[30] Lee JS (2019). PCSK9 is increased in cerebrospinal fluid of individuals with alcohol use disorder. Alcohol Clin. Exp. Res..

[31] Momtazi-Borojeni AA (2019). PCSK9 and inflammation: a review of experimental and clinical evidence. Eur. Heart J. Cardiovasc. Pharmacother..

[32] Shafabakhsh R, Reiner Z, Hallajzadeh J, Mirsafaei L, Asemi Z (2020). Are anti-inflammatory agents and nutraceuticals—novel inhibitors of PCSK9?. Crit. Rev. Food Sci. Nutr..

[33] Bajaj NS (2018). Neurological effects of proprotein convertase subtilisin/kexin type 9 inhibitors: direct comparisons. Eur. Heart J. Qual. Care Clin. Outcomes.

[34] de Melo LGP (2017). Shared metabolic and immune-inflammatory, oxidative and nitrosative stress pathways in the metabolic syndrome and mood disorders. Prog. Neuropsychopharmacol. Biol. Psychiatry.

[35] Pandey GN (2017). Inflammatory and innate immune markers of neuroprogression in depressed and teenage suicide brain. Mod. Trends Pharmacopsychiatry.

[36] Byrnes SA, Weigl BH (2018). Selecting analytical biomarkers for diagnostic applications: a first principles approach. Expert Rev. Mol. Diagn..

[37] Endicott J, Spitzer RL (1978). A diagnostic interview: the schedule for affective disorders and schizophrenia. Arch. Gen. Psychiatry.

[38] Benazzi F (2007). Testing predictors of bipolar-II disorder with a 2-day minimum duration of hypomania. Psychiatry Res..

[39] Angst J (2003). Toward a re-definition of subthreshold bipolarity: epidemiology and proposed criteria for bipolar-II, minor bipolar disorders and hypomania. J. Affect. Disord..

[40] Young RC, Biggs JT, Ziegler VE, Meyer DA (1978). A rating scale for mania: reliability, validity and sensitivity. Br. J. Psychiatry.

[41] Hamilton M (1960). A rating scale for depression. J. Neurol. Neurosurg. Psychiatry.

[42] Hamilton M (1967). Development of a rating scale for primary depressive illness. Br. J. Soc. Clin. Psychol..

[43] Sharma R (2015). Proteomic signature of endothelial dysfunction identified in the serum of acute ischemic stroke patients by the iTRAQ-based LC–MS approach. J. Proteome Res..

[44] Berk M (2011). Pathways underlying neuroprogression in bipolar disorder: focus on inflammation, oxidative stress and neurotrophic factors. Neurosci. Biobehav. Rev..

[45] Zadjali F (2018). Homozygosity for FARSB mutation leads to Phe-tRNA synthetase-related disease of growth restriction, brain calcification, and interstitial lung disease. Hum. Mutat..

[46] Avramopoulos D (2015). Infection and inflammation in schizophrenia and bipolar disorder: a genome wide study for interactions with genetic variation. PLoS ONE.

[47] Faul F, Erdfelder E, Buchner A, Lang A (2009). Statistical power analyses using G*Power 3.1: tests for correlation and regression analyses. Behav. Res. Methods.

