# Supplementary material for: Identification of potential plasma protein biomarkers for bipolar II disorder: a preliminary/exploratory study
Source: Sci Rep. 2021 May 4;11:9452. doi: 10.1038/s41598-021-88450-x (PMC8097016; doi:10.1038/s41598-021-88450-x)
Supplement: Supplementary file 1 — Supplementary Information 1. [file 41598_2021_88450_MOESM1_ESM.docx]

**Supplement Table 1. Differences in proteins between male and female in BD-II and Control groups**

|  | t | P |
| --- | --- | --- |
| BD-II group |  |  |
| PRDX2 | 3.598 | <0.001** |
| CA1 | 4.182 | <0.001** |
| FARSB | 1.920 | 0.057 |
| MMP9 | 4.040 | <0.001** |
| PCSK | -1.303 | 0.195 |
| Controls group |  |  |
| PRDX2 | 0.906 | 0.366 |
| CA1 | 2.365 | 0.019* |
| FARSB | 2.107 | 0.037* |
| MMP9 | 2.513 | 0.013* |
| PCSK | -1.748 | 0.082 |
