# Supplementary material for: Identification of potential plasma protein biomarkers for bipolar II disorder: a preliminary/exploratory study
Source: Sci Rep. 2021 May 4;11:9452. doi: 10.1038/s41598-021-88450-x (PMC8097016; doi:10.1038/s41598-021-88450-x)

**Identification of potential plasma protein biomarkers for bipolar II disorder: A preliminary/exploratory study**

*Sheng-Yu Lee, MD, PhD^1,2^, Tzu-Yun Wang, MD^3^, Ru-Band Lu, MD^3,4^, Liang-Jen Wang, MD, MPH, PhD^5^, Sung-Chou Li, PhD^6^, Chi-Ying Tu*, *ＭS^7^, Cheng-Ho Chang, MD, PhD^1^, Yung-Chih Chiang, MD^1^, Kuo-Wang Tsai, PhD^8^*

^1^Department of Psychiatry, ^7^Department of Medical Education and Research, Kaohsiung Veterans General Hospital, Kaohsiung, Taiwan; ^2^Department of Psychiatry, Faculty of Medicine, Kaohsiung Medical University Kaohsiung, Taiwan; ^3^Department of Psychiatry, National Cheng Kung University Hospital, College of Medicine, National Cheng Kung University, Tainan, Taiwan; ^4^Yanjiao Furen Hospital, Hebei, China; ^5^Department of Child and Adolescent Psychiatry, ^6^Genomics and Proteomics Core Laboratory, Department of Medical Research, Kaohsiung Chang Gung Memorial Hospital and Chang Gung University College of Medicine, Kaohsiung, Taiwan; ^8^Department of Research, Taipei Tzu chi Hospital, Buddhist Tzu chi Medical Foundation, New Taipei, Taiwan

**Running title:** Plasma protein as a biomarker for bipolar II

**Word counts:** Abstract = 198 words; text = 3817 words; 3 tables. 2 figures and 2 supplementary figures, 1 supplementary table

**Correspondence:**

Kuo-Wang Tsai, PhD

Assistant Professor

Department of Research,

Taipei Tzu Chi Hospital,

The Buddhist Tzu Chi Medical Foundation,

New Taipei, Taiwan.

E-mail: kwtsai6733@gmail.com

**Figure Legends**

**Supplement Figure 1.** The top 38 differential expressed protein candidates (19 with upregulation and 19 with downregulation) presented with heatmap.

**Supplement Figure 2(a) and (b).** Upregulated or downregulated proteins candidates subjected to gene ontology analysis.

Supplement Figure 1.


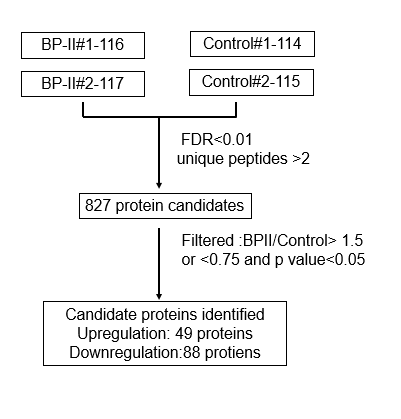


Supplement Figure 2(a) and (b).

2(a)


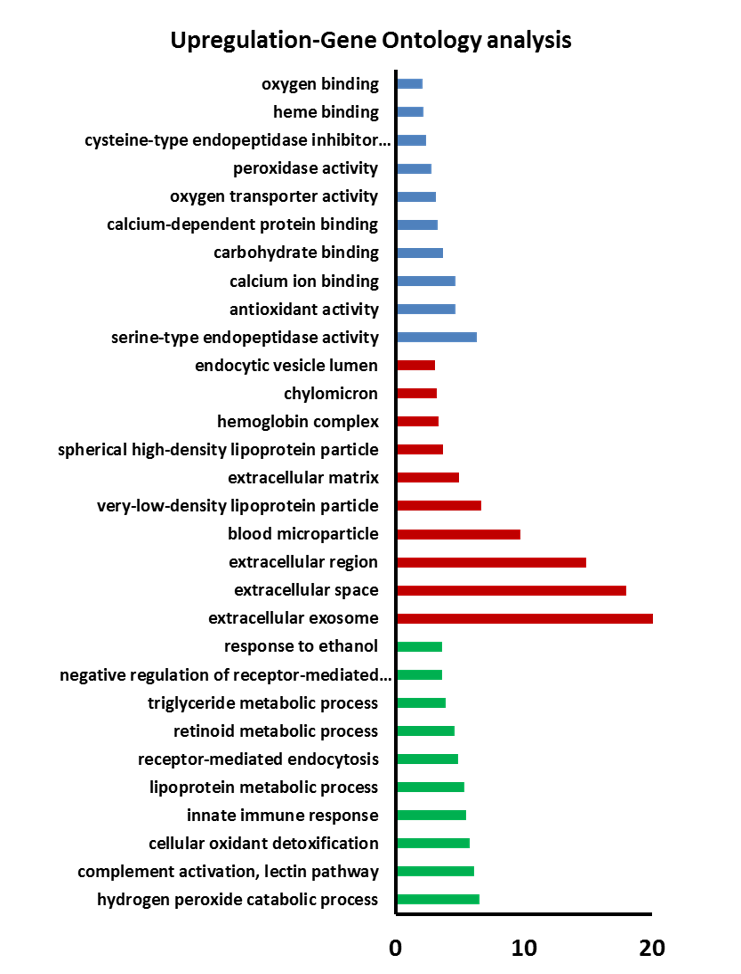


2(b)


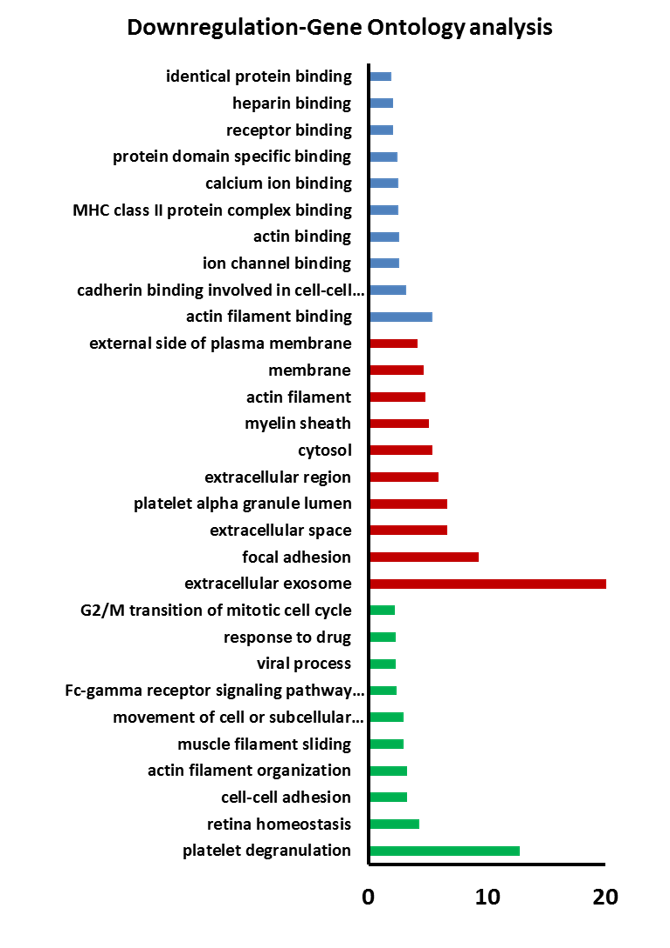

Supplement: Supplementary file 2 — Supplementary Information 2. [file 41598_2021_88450_MOESM2_ESM.docx]
